# Persistent Contamination of Octopuses and Mussels with Lipophilic Shellfish Toxins during Spring *Dinophysis* Blooms in a Subtropical Estuary

**DOI:** 10.3390/md13063920

**Published:** 2015-06-18

**Authors:** Luiz L. Mafra, Daiana Lopes, Vanessa C. Bonilauri, Hajime Uchida, Toshiyuki Suzuki

**Affiliations:** 1 Center for Marine Studies, Federal University of Paraná, P.O. Box 61, Pontal do Paraná, Paraná 83255-976, Brazil; E-Mails: daiana.aquicultura2011@gmail.com (D.L.); vancoq@ufpr.br (V.C.B.); 2 National Research Institute of Fisheries Science, 2-12-4 Fukuura, Kanazawa, Yokohama, Kanagawa 236-8648, Japan; E-Mails: huchida@affrc.go.jp (H.U.); tsuzuki@affrc.go.jp (T.S.)

**Keywords:** diarrheic shellfish poisoning, toxin accumulation, tissue distribution, *Octopus vulgaris*, *Perna perna*, *Dinophysis acuminata* complex

## Abstract

This study investigates the occurrence of diarrhetic shellfish toxins (DSTs) and their producing phytoplankton species in southern Brazil, as well as the potential for toxin accumulation in co-occurring mussels (*Perna perna*) and octopuses (*Octopus vulgaris*). During the spring in 2012 and 2013, cells of *Dinophysis acuminata* complex were always present, sometimes at relatively high abundances (max. 1143 cells L^−1^), likely the main source of okadaic acid (OA) in the plankton (max. 34 ng L^−1^). *Dinophysis caudata* occurred at lower cell densities in 2013 when the lipophilic toxins pectenotoxin-2 (PTX-2) and PTX-2 seco acid were detected in plankton and mussel samples. Here, we report for the first time the accumulation of DSTs in octopuses, probably linked to the consumption of contaminated bivalves. *Perna perna* mussels were consistently contaminated with different DSTs (max. 42 µg kg^−1^), and all octopuses analyzed (*n* = 5) accumulated OA in different organs/tissues: digestive glands (DGs) > arms > gills > kidneys > stomach + intestine. Additionally, similar concentrations of 7-*O*-palmytoyl OA and 7-*O*-palmytoly dinophysistoxin-1 (DTX-1) were frequently detected in the hepatopancreas of *P. perna* and DGs of *O. vulgaris*. Therefore, octopuses can be considered a potential vector of DSTs to both humans and top predators such as marine mammals.

## 1. Introduction

Accumulation of Diarrhetic Shellfish Toxins (DSTs) in bivalve mollusks has caused expressive economic losses and human intoxication outbreaks in different parts of the globe (reviewed in Reguera *et al*. [1]). DSTs are acidic polyethers that may inhibit serine/threonine phosphoprotein phosphatases [2], causing diarrhea and other gastrointestinal symptoms in humans [3] during outbreaks of a food intoxication known as Diarrhetic Shellfish Poisoning (DSP). Since the late 1970s [4,5], DSP episodes have been reported worldwide following the consumption of contaminated bivalve mollusks, as assessed mainly by mice bioassay (MBA) and, more recently, by liquid chromatography-mass spectrometry (LC-MS) techniques [1]. The causative toxins, okadaic acid (OA) and its relative compounds, the dinophysistoxins (DTXs), can provoke not only phosphatase inhibition thus leading to DSP symptoms, but also immunotoxicity, genotoxicity and cytotoxicity to many cell types, (reviewed in Valdiglesias *et al.* [6]), and even tumor formation upon chronic exposure [7].

Okadaic acid and their analogs, DTX-1 and DTX-2, are produced by 10 species of the dinoflagellate *Dinophysis*, along with a few other species of the genera *Phalacroma* and *Prorocentrum*, which may retain varying concentrations of DSTs in their cells [1]. Among the toxin producers, the taxonomic complex composed by *Dinophysis acuminata*, *Dinophysis ovum*, and *Dinophysis sacculus*, the so-called *D. acuminata* complex, is the most widespread *Dinophysis* group in coastal areas [8] and is responsible for most DSP episodes. In addition, some *Dinophysis* species may produce another group of lipophilic shellfish toxins (LSTs), the pectenotoxins (PTXs), which are commonly found in plankton and bivalve tissues during DSP events and may interfere with MBA results, although the toxicity of these polyether-lactones to humans is uncertain because they are hepatotoxic to mice by intra-peritoneal injection but not by oral administration [9].

The amount of toxins accumulated by marine organisms will depend on the abundance of toxic phytoplankton cells they are exposed to. Even though *Dinophysis* spp. are usually minor components of mixed phytoplankton assemblages, cell densities as low as 100–200 cells L^−1^ may be enough to cause DSP outbreaks [3] if the cell toxin quota is sufficiently high. Moreover, feeding characteristics of the contaminated organisms, such as particle capture efficiency, capacity for selective feeding, ingestion rates, digestion efficiency, affinity for the toxic compounds, toxin transformation (*i.e.*, metabolism and conjugation), and excretion [10], as well as their position in the water column [11], will ultimately determine the degree of toxin accumulation and its trophic transfer following contact with toxic *Dinophysis* cells in the water. Bivalves are the main vector for human intoxication, but other organisms, including polychaetes, ascidians [11], crabs [12] and fishes [13,14], have demonstrated the capacity to accumulate low DST levels following the ingestion of either toxic cells or contaminated bivalves.

There is currently no report of DST accumulation in octopuses, although they actively prey upon bivalves and may accumulate other shellfish toxins, such as the amnesic toxin domoic acid [15,16] and paralytic toxins [17,18,19,20].

The present study investigates the presence of DSTs and other LSTs in the plankton and mussels (*Perna perna*) at two depths of a marina area in the outer Paranaguá Bay, southern Brazil, as well as in mussel-feeding octopuses (*Octopus vulgaris*) that reside that same area. Over two consecutive years (2012–2013), samples were obtained during the spring months, a period of typically higher cell densities of *D. acuminata* complex in the region. The occurrence of DSTs and other LSTs was evaluated in multiple tissues/organs of both mussels and octopuses to assess the risk for chronic LST exposure among human consumers and marine predators.

## 2. Results and Discussion

### 2.1. Cell Abundance and Toxin Concentration in the Plankton

**Figure 1:**
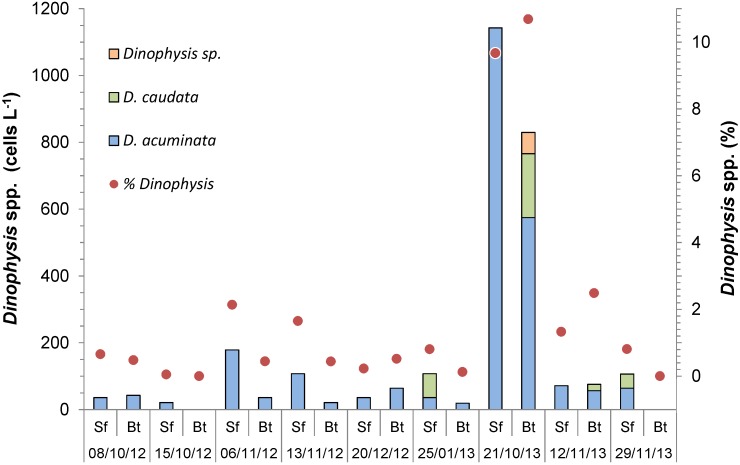
Cell density (cells L^−1^) and the relative abundance (% of total microphytoplankton) of *Dinophysis* spp. in each sample collected from a marina area at the outer portion of Paranaguá Bay, southern Brazil. Sf: surface; Bt: bottom.

*D. acuminata* was present in most (89%) plankton samples, usually at cell densities <100 cells L^−1^. In October 2013, however, a maximum of 1143 cells L^−1^ was registered in the sampling area. During the same period, the occurrence of a *Dinophysis* species resembling *D. parvula* (64 cells L^−1^) and the maximum cell density of *D. caudata* (192 cells L^−1^) were also detected (Figure 1). In fact, *D. caudata* was only found in the area in 2013. *Dinophysis* spp. usually represented a small portion (<2.5%) of the total microphytoplankton cell abundance, except in October 2013, when approximately 10% of the total abundance (9.7% at the surface and 10.7% near the bottom) consisted of *Dinophysis* cells (Figure 1). Cell densities as low as 100–200 cells L^−1^ may be sufficient to cause DSP outbreaks [3], especially if *Dinophysis* relative abundance and cell toxin quota are high.

**Figure 2:**
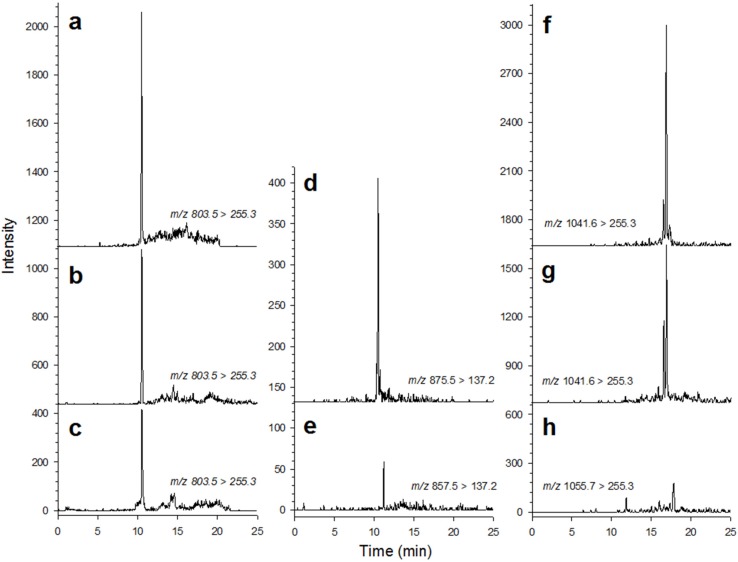
Multiple reaction monitoring (MRM) LC-MS/MS chromatograms of (**a**–**c**) okadaic acid (OA); (**e**) pectenotoxin-2 (PTX-2); (**d**) PTX-2 seco acid; (**f**,**g**) 7-*O*-palmytoyl OA and (**h**) 7-*O*-palmytoyl dinophysistoxin-1 (DTX-1) in selected samples of: (**a**,**d**,**g**) hepatopancreas of *Perna perna* mussels, (**b**,**e**) plankton, and (**c**) arms and (**f**,**h**) digestive glands of *Octopus vulgaris* from Paranaguá Bay, southern Brazil.

**Figure 3:**
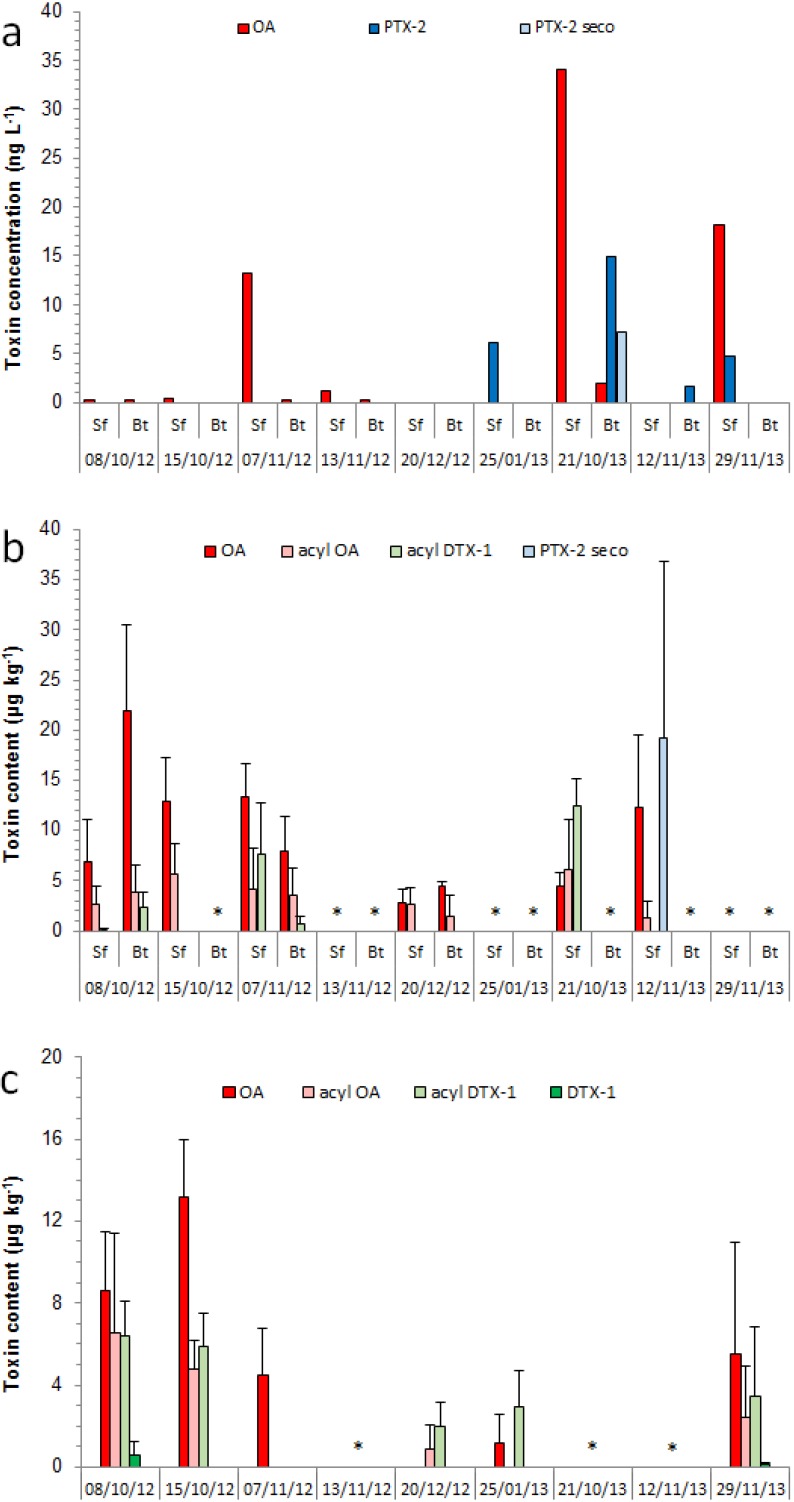
Toxin contents (mean + standard error) in (**a**) plankton; (**b**) whole mussels (reconstituted samples) and (**c**) octopus digestive glands sampled in Paranaguá Bay, southern Brazil. Asterisks indicate occasions when no samples were available. Sf: surface; Bt: bottom.

Different LSTs were detected in plankton, mussels and octopus samples (Figure 2). The highest OA concentrations in the plankton were measured at the surface, reaching 13.2 ng L^−1^ on 7 November 2012, 18.1 ng L^−1^ on 29 November 2013 and the maximum value of 34.1 ng L^−1^ on 21 October 2013 (Figure 3a), coincident with the peaks in *D. acuminata* cell abundance. Average OA cell quotas ranged from 2.3 to 19.2 pg OA *Dinophysis* cell^−1^, which is within the normal range reported for the region (2.4–26.4 pg OA cell^−1^ [14]).

*D. acuminata* complex was either the only (spring 2012) or the dominant (spring 2013) *Dinophysis* present in the samples, likely to be responsible for the OA found in the water column due to its overwhelming presence. In fact, species belonging to the *D. acuminata* complex are the main culprit for DSP episodes across the globe (reviewed in Reguera *et al*. [1]), although their toxin profile and cell quota may vary at a great extent according to the geographic location. In culture, LST cell quotas range from non-detected levels to 0.9 pg OA cell^−1^, 1.8 pg DTX-1 cell^−1^ and 20.4 pg PTX-2 cell^−1^ in isolates from northwestern Atlantic [21,22,23,24]; and from non-detected levels to 12.2 pg OA cell^−1^, from 0.2 to 4.8 pg DTX-1 cell^−1^ and from 14.7 to 107 pg PTX-2 cell^−1^ in isolates from Japan [25,26,27]. In some other areas, isolates belonging to the *D. acuminata* complex, including *D.* cf. *ovum*, may produce only OA, as reported in the Gulf of Mexico (12.6 pg cell^−1^ [22,24]) and southern Brazil (3.2–18 pg cell^−1^ [14]).

During the 2013 bloom season, PTX-2 and PTX-2 seco acid were also detected in the plankton, coinciding with the presence of *D. caudata* in the water, and attained, respectively, up to 11.2 and 5.4 ng L^−1^ near the bottom on 21 November 2013 (Figure 3a).

### 2.2. Toxin in Mussels and Octopuses

Mussels were contaminated with OA (max. 21.9 ± 8.6 µg kg^−1^) whenever samples were available during the study. In addition, 7-*O*-palmytoyl OA and 7-*O*-palmytoyl DTX-1 were occasionally found at lower concentrations (max. 6.1 ± 4.9 and 12.4 ± 2.8 µg kg^−1^, respectively) (Figure 3b). Concentrations of DSTs in mussels were similar between sampling depths and among sampling campaigns, and there was no clear association with maximum OA levels measured in the plankton. Conversely, PTX-2 seco acid was detected in mussels in a single occasion, following the period when the maximum levels of PTX-2 were found in the plankton. OA was found more frequently (96%) and at higher levels (up to 69.7 µg kg^−1^) in the hepatopancreas (HP) compared to the non-visceral (NV) tissues of mussels (42%; max. 4.0 µg kg^−1^) (Table 1). On average, mussels accumulated 10 times higher OA levels in the HP than in the NV tissues. The metabolites 7-*O*-palmytoyl OA, 7-*O*-palmytoyl DTX-1 and PTX-2 seco acid were detected exclusively in the HP (Table 1), along with a different DTX-1 analogue (not shown), which exhibited the characteristic *m*/*z* 255.1 fragment, but slightly different MS/MS spectrum and retention time (10.5 min) than that usually obtained for DTX-1 (11.7 min). Additional MS/MS and NMR analysis will be conducted in order to elucidate the molecular structure of this novel DTX-1 analogue.

**Table 1:** Average (avg., ± standard deviation) and maximum (max.) toxin levels measured in different tissues/organs of mussels *Perna perna* sampled in Paranaguá Bay, southern Brazil. Number of positive samples and the total number of samples analyzed (*n*) are indicated in parenthesis. Each sample was composed of four pooled individuals. nd: non-detected.

Tissue		OA		acyl OA		acyl DTX-1		PTX-2 seco	
		µg kg^−1^	*n*	µg kg^−1^	*n*	µg kg^−1^	*n*	µg kg^−1^	*n*
Hepatopancreas	avg.	22.2 ± 17.9	(25/26)	8.87 ± 10.3	(17/26)	7.81 ± 15.2	(10/26)	6.34 ± 27.2	(2/26)
	max.	69.7		43.2		53.3		137.0	
Non-visceral tissues	avg.	0.94 ± 1.30	(11/26)	nd		nd		nd	
	max.	4.04							

Although mussels did not accumulate DTX-1 at detectable levels, 37% of the *P. perna* samples exhibited low levels of its acylated derivative, 7-*O*-palmytoyl DTX-1 (up to 16.9 µg kg^−1^), and 65% also presented low levels of 7-*O*-palmytoyl OA (up to 13.7 µg kg^−1^). The maximum total DST levels were approximately four-fold lower than the EU regulatory limit (RL) of 160 ng OA equiv. g^−1^ [28], and similar to those found in oysters *Crassostrea* spp. sampled in January 2011 from the same region (up to 17.8 µg kg^−1^ [14]). In contrast, in neighboring areas of Santa Catarina State, *P. perna* mussels and, eventually, the oyster *Crassostrea gigas* have accumulated OA levels above the RL when exposed to similar to higher *Dinophysis* cell densities (*i.e.*, >1000 cells L^−1^), leading to DSP outbreaks on some occasions [29,30].

In addition to DSTs, mussels sampled in the present study also accumulated PTX-2 seco acid, a common degradation product of PTX-2 in bivalves [9,31]. Since southern Brazilian populations of *D. acuminata* complex apparently produce only OA [14], *D. caudata* is probably the sole source of PTX-2 detected in the present study. The highest observed *D. caudata* cell abundances (~190 cells L^−1^) coincided with the maximum PTX-2 concentrations (up to 11.2 ng L^−1^) reported in the plankton fraction in late November 2013. Assuming no other producer, average PTX-2 contents ranged from 58.2 to 112 pg *D. caudata* cell^−1^. Moreover, the presence of PTX-2 seco acid in one of our plankton samples must be related to the ingestion (and further digestion) of *D. caudata* cells by zooplankton. Although *D. caudata* has been previously cultivated in the laboratory [14,32], the only reported toxin profile (7.9–56.5 pg OA; 0.0–53.9 pg DTX-1 cell^−1^) was obtained from picked wild cells in the Philippines, when no PTX analysis was performed [33]. Recently, PTX-2 was the only LST detected in the dissolved fraction of a *D. caudata* culture isolated from Japanese waters [34], supporting the indication of this species as an important source of PTX-2 in southern Brazil, what remains to be unambiguously confirmed and quantified in the field.

In November 2013, toxin concentrations reached 34.1 ng OA L^−1^ and 11.2 ng PTX-2 L^−1^ in our plankton samples, when the cell densities of both *D. acuminata* complex and *D. caudata* were the highest (1140 and 190 cells L^−1^, respectively). In a similar study in Jinhae Bay, Korea, equivalent concentrations of PTX-2 (8.1 ng L^−1^) but much lower levels of OA and DTX-1 (0.8 ng L^−1^ each) were detected in the plankton when *D. acuminata* reached 1000 cells L^−1^ [35]. Notwithstanding, mussels *Mytilus galloprovincialis* from Jinhae Bay accumulated relatively higher LST levels in their HPs (up to 168 ng OA g^−1^, 171 ng DTX-1 g^−1^ and 105 ng PTX-2 g^−1^) compared to those found in *P. perna* in the present study (Table 1), suggesting an inferior capacity for toxin accumulation in the latter. In *M. galloprovincialis*, LST levels varied accordingly to both *Dinophysis* cell abundance and toxin concentrations in the plankton [35]. However, this was not always the case with *P. perna* mussels in the present study (see 2012 data in Figure 1 and Figure 3), what may be related to their much faster OA detoxification rates (0.023 h^−1^), as calculated during simulated *Dinophysis* blooms in the laboratory [30]. In addition, 7-*O*-palmytoylated derivatives of OA and DTX-1 attained slightly greater concentrations than those of OA in the HPs of *P. perna*, suggesting fast toxin transformation and elimination in that organ. When present in the other soft tissues, OA concentrations were, on average, 10-fold (3.9–26-fold) lower than those measured in the HPs.

Besides being harvested for human consumption, mussels contaminated with low to moderate toxin concentrations can be consumed by a wide range of marine benthic predators, such as fish [13], crustaceans [36], gastropods [37] and cephalopods [18]. At that point, these organisms may become intoxicated and/or act as a vector of toxins to higher trophic levels. In the present study, LST-contaminated mussels living on the columns of a wharf may have served as an important food item for a local population of common octopuses, *Octopus vulgaris*, as evidenced by the presence of abundant empty shells around their sheltering holes.

All five octopuses sampled during this study accumulated toxins in at least two different organs/tissues each. The highest OA concentrations were detected in the digestive glands (DGs; max. 17.3 µg kg^−1^) of a male individual (1130 g; 14.1 cm mantle length, ML) sampled on 15 November 2012 (Figure 3c), and in the gills (max. 13.2 µg kg^−1^) and arms (max. 43.7 µg kg^−1^) of a smaller male (870 g; 13.5 cm ML) sampled on 20 December 2012. Lower toxin levels were reported in the DGs (max. 7.9 µg kg^−1^), gills (max. 8.0 µg kg^−1^) and kidneys (max. 7.2 µg kg^−1^) of two female individuals sampled on 7 November 2012 and 25 January 2013 (1450 and 890 g; 16.4 and 14.9 cm ML, respectively). In addition to OA, 7-*O*-palmytoyl OA (max. 14.4 µg kg^−1^) and 7-*O*-palmytoyl DTX-1 (max. 8.8 µg kg^−1^) were also found in the DGs of different octopuses, and DTX-1 and 7-*O*-palmytoyl DTX-1 were detected, respectively, in the DG and the stomach + intestine fraction of a large male (2220 g; 14.9 cm ML) sampled on 8 November 2012 (Figure 3c, Table 2). Finally, 7-*O*-palmytoyl OA was also found at low levels in the stomach + intestine, gills and kidneys of the octopus sampled on 15 November 2012 (Table 2), while PTX-2 and PTX-2 seco acid were not detected in any of the five octopuses. DTX-2 and PTX-6 were not found in any sample analyzed during this study, including plankton, mussels and octopuses.

**Table 2:** Average (avg., ± standard deviation) and maximum (max.) toxin levels measured in different tissues/organs of octopuses *Octopus vulgaris* sampled in Paranaguá Bay, southern Brazil. Number of contaminated individuals and the total number of individuals analyzed (*n*) are indicated in parenthesis. nd: non-detected.

		OA		acyl OA		acyl DTX-1		DTX-1	
Tissue		ng g^−1^	*n*	ng g^−1^	*n*	ng g^−1^	*n*	ng g^−1^	*n*
Digestive gland	avg.	5.49 ± 5.66	(4/5)	2.44 ± 3.94	(3/5)	3.41 ± 3.02	(4/5)	0.11 ± 0.44	(1/5)
	max.	17.3		14.4		8.8		1.7	
Stomach + intestine	avg.	0.43 ± 1.70	(1/5)	0.06 ± 0.23	(1/5)	0.04 ± 0.16	(1/5)	nd	
	max.	6.6		0.9		0.6			
Gills	avg.	2.48 ± 3.94	(3/5)	0.02 ± 0.09	(1/5)	nd		nd	
	max.	13.2		0.4					
Kidneys	avg.	1.12 ± 2.48	(2/5)	0.10 ± 0.33	(1/5)	nd		nd	
	max.	7.3		1.0					
Arm	avg.	3.72 ± 11.3	(2/5)	nd		nd		nd	
	max.	43.7							
Gonads	avg.	nd		nd		nd		nd	
	max.								
Mantle	avg.	nd		nd		nd		nd	
	max.								

Thus, based on the present findings in Southern Brazil, octopuses can be considered an additional vector of these toxins to both humans and top predators such as marine mammals, known to prey upon octopuses and other cephalopods [38,39]. In fact, this may be also relevant in other regions worldwide, since both LST-producing dinoflagellates [40] and *O. vulgaris* [41] are globally distributed in tropical to temperate waters, and these octopuses are able to retain large amounts of biocumulative substances like heavy metals (e.g., [42,43]) and other algal toxins, such as paralytic shellfish toxins (PSTs), domoic acid (DA) and palytoxins (PlTXs) (reviewed in Lopes *et al*. [44]).

Octopuses, including an unidentified species from Australia and the common octopus *O. vulgaris*, have shown the capacity to accumulate large amounts of PSTs [17,18,19,20]. Likewise, *O. vulgaris* and two other octopus species from the Portuguese continental coast, *Eledone cirrhosa* and *E. moschata*, were found to contain relatively high concentrations of the amnesic shellfish toxin, domoic acid (DA) [15,16]. By far, the highest PST and DA levels are observed in the octopus DGs. *O. vulgaris*, for instance, accumulated up to 2680 ng STX equiv. g^−1^ in the DG following the consumption of contaminated mussels during toxic blooms in Portugal, while other organs involved in digestion (stomach and salivary glands) and excretion (kidneys and branchial hearts) exhibited approximately eight to 16 times lower toxin concentrations [19]. This was further confirmed under laboratory conditions, with *O. vulgaris* accumulating up to three orders of magnitude higher PST levels in the DG compared to the kidneys [20]. Finally, DA concentrations in the DG of Portuguese *O. vulgaris* were approximately three times higher than those measured in the branchial hearts, and one to two orders of magnitude higher than those eventually found in the kidneys, digestive tract (stomach, spiral caecum and intestine), gills, systemic heart, posterior salivary glands and mantle [15]. In the present study, the tissue distribution of LSTs varied among the five individuals investigated, however, total toxin levels (OA + DTX-1 + acyl OA + acyl DTX-1) were, on average, much higher in the DG (11.5 µg kg^−1^) than in the arms (3.7 µg kg^−1^), gills (2.5 µg kg^−1^), kidneys (1.2 µg kg^−1^) and stomach + intestine (0.5 µg kg^−1^). Toxin levels in mantle and gonads were always below the detection limit (*i.e.*, 0.11 µg kg^−1^).

The higher LST levels detected in the DGs of *O. vulgaris* support their role as the primary site for intracellular digestion, absorption and storage in cephalopods [45]. In four out of five octopuses collected in the present study, DG samples contaminated with OA also contained the acylated metabolites 7-*O*-palmytoyl OA and/or 7-*O*-palmytoyl DTX-1, indicating active LST transformation at that organ. Acylated derivatives were much less frequent in other octopus tissues where OA was detected at lower levels, such as the gills, suggesting that a limited fraction of the toxin may enter the blood system and be distributed prior to its absorption by the digestive system. In addition, toxin detection in the kidneys may indicate that these organs are involved in excretion of LSTs by octopuses, as first suggested for PSTs [19] and DA [15].

Surprisingly, OA was also detected in the arms of two octopuses in our study, reaching relatively high yet extremely variable toxin levels (CV = 124%–173%). For instance, OA levels varied from <1.0 to 43.7 µg kg^−1^ within a single arm. Since our replicate samples were composed of different pieces of tissue from a single octopus arm, this suggests strong spatial toxin aggregation into putative high affinity sites, as reported for saxitoxin (STX) in the siphons of butter clams, *Saxidomus giganteus* ([46] and references therein). In the case of octopus, this may be related to the practice of discarding the very tip of the arms in some traditional Western Japanese cuisine supposedly avoiding consumer intoxication by bacteria and marine toxins, although we cannot trace back which part of the octopus arms contained the higher toxin levels in our study. In fact, STX has been found at relatively high levels in the arms of an unidentified octopus from Australia [18], but never in *O. vulgaris*, despite the extremely high toxin contents measured in their DGs [19]. The possibility that octopuses may also accumulate low toxin levels in other organ/tissues not related to digestion and excretion, such as the mantle and gonads, cannot be disregarded and should be further assessed in laboratory-based toxin dynamics studies, and/or during more toxic episodes of *Dinophysis* natural blooms.

## 3. Experimental Section

### 3.1. Field Sampling

Nine sampling campaigns were performed from 8 October 2012 to 25 January 2013 and from 21 October to 29 November 2013 at a marina area in the outer sector of Paranaguá Bay, southern Brazil (25°32′82.2′′ S; 48°23′28.3′′ W). *P. perna* mussels colonize the columns of a wharf located in this area and are used as part of the diet of a resident *O. vulgaris* population [47].

Phytoplankton samples were always taken at flood to high tide using Van Dorn bottles at sub-surface (0.5 m) and near the bottom (9–9.5 m depth) for cell density determination and toxin analysis. Samples were fixed with 1% neutral Lugol’s solution, and *Dinophysis* spp. cells were counted on entire Utermöhl chambers under the microscope, after sedimentation of 50 mL. Other microphytoplankton cells (>20 μm) were counted in transects until at a minimum of 300 cells had been computed. *Dinophysis* spp. cell density was expressed as cells L^−1^ and the relative abundance as percent of total microphytoplankton cells, for both surface and bottom samples. Concurrent to bottle sampling, oblique net trawling (20 µm mesh size) was performed for taxonomic identification. *Dinophysis* cells were observed under the light microscope (Zeiss^®^ Axiovert A1), and randomly selected individuals were measured using a coupled camera (Zeiss^®^ AxioCam ERc5s) and the image processing software AxioVision 4. Cell features, shape and measurements were compared to the literature [48,49,50] for taxonomic identification at species level.

Aliquots (100 mL) of the bottle samples were gently passed through glass microfiber filters (Whatman^®^ GF/F) and the retained material was kept frozen for further toxin analysis. In addition, 100-mL aliquots of the net concentrated samples were also filtered for toxin determination in the case toxin levels in plankton were below the detection limit. In this case, triplicate 2–25 mL aliquots of the net-concentrated samples were settled, and *Dinophysis* spp. cells were counted on entire chambers, as previously described, in order to calculate the average cellular toxin content (expressed in pg cell^−1^).

Mussels and octopuses were simultaneously sampled by scuba diving during the plankton sampling campaigns. Mussels were manually removed from the wharf columns at sub-surface and near the bottom, and immediately transported to the laboratory. After external cleaning, mussel shells were opened and the market-sized individuals were carefully dissected into hepatopancreas (HP) and non-visceral (NV) soft tissues. Samples (*n* = 3 per depth) were composed of pooled tissue fractions from four mussels, yielding at least 5 g of HP or 10 g of NV tissues each, and individually frozen until further processing. Octopuses, when available, were collected from their holes near the wharf and frozen upon arrival at the laboratory. Prior to the toxin analysis, adult individuals were dissected during thawing to prevent leaking of fluids and contamination of surrounding organs. Aliquots (*n* = 3; 1–5 g each) of the following tissues/organs were obtained and kept frozen into individual tubes for further analysis: digestive glands, stomach + intestine, gills, kidneys, gonads, arms and mantle. Only two replicate samples were obtained from the kidneys due to their reduced size.

### 3.2. Toxin Analysis

Toxins were extracted from the plankton samples after adding 5 mL of 100% methanol (JT Baker, Phillipsburg, NJ, USA) to each filter. Plankton cells retained in the filters were disrupted using a sonic dismembrator (Cole Parmer CPX130, Vernon Hills, IL, USA), applying pulses of 2 s with 1-s intervals for 2 min, at 70% amplitude. Extracts were then centrifuged for 10 min at 2400 rpm; a 0.5-mL aliquot of the supernatant was completely evaporated with nitrogen and stored in freezer for further toxin analysis, when the same volume of methanol was used to reconstitute the samples. Bivalve and octopus tissues were processed following a similar protocol, except that methanol was added on a 9 mL:1 g proportion, with the sonicator set at 80% amplitude, applying pulses of 3 s and intervals of 1 s for 3 min. Samples of octopus mantle and arms were first ground with an Ika Ultra-Turrax^®^ disperser.

Toxin analysis was conducted by liquid chromatography-tandem mass spectrometry (LC-MS/MS) using an Agilent 1200 series (Agilent Technologies, Palo Alto, CA, USA) LC system coupled to a hybrid triple quadrupole/linear ion trap mass spectrometer Q-trap^®^ 3200 (AB-SCIEX, Framinghan, MA, USA) equipped with a TurboIonSpray interface. LC-MS/MS analysis was carried out according to the method described in Suzuki *et al*. [51], with a slight modification—the use of multiple reaction monitoring (MRM) instead of selected ion monitoring (SIM) [52]. Separations were performed on a Quicksilver cartridge column (50 mm × 2.1 mm i.d.) packed with 3 µm Hypersil-BDS-C8 (Thermo Fisher Scientific, Waltham, MA, USA) maintained at 20 °C. Both eluent A (water) and B (95% acetonitrile) contained 2 mM ammonium formate and 50 mM formic acid [53,54]. Linear gradient elution from 20% to 100% B was performed over 10 min and then held at 100% B for 15 min, followed by re-equilibration with 20 % B for 13 min. Flow rate was 0.2 mL min^−1^ and the injection volume 5 µL. The LC effluent was introduced into the TurboIonSpray interface without splitting. High-purity air, heated to 500 °C, was used as the nebulizing gas. The following SRM transitions were monitored: *m*/*z* 803.5→255.1 for OA and DTX-2; *m*/*z* 817.5→255.1 for DTX-1; *m*/*z* 1041.6→255.3 for 7-*O*-palmytoyl OA, *m*/*z* 1055.7→255.3 for 7-*O*-palmytoly DTX-1, *m*/*z* 857.5→137.2 for PTX-2, *m*/*z* 875.5→137.2 for PTX-2 seco acid, *m*/*z* 873.5→137.2 for PTX-1 and *m*/*z* 887.5→519.4 for PTX-6 [53], with OA, DTX-1 and PTX-2 concentrations calculated from a calibration curve made of serial dilutions of the reference standards available at FRA-NRIFS, Yokohama, Japan, using the software Analyst^®^. The calculated detection limits were 0.011, 0.018 and 0.030 ng mL^−1^ for OA, DTX-1 and PTX-2, respectively.

## 4. Conclusions

This is the first report of DST accumulation in the common octopus (*O. vulgaris*), probably linked to the consumption of contaminated bivalves during periods when *Dinophysis* spp. were persistently present at moderate cell abundances in the water. All five octopuses analyzed in the present investigation accumulated DSTs in different organs/tissues, including their edible arms. Likewise, mussels sampled near the octopus shelters were consistently contaminated, containing low to moderate DST levels.

Over the sampling period (spring seasons of 2012 and 2013), *D. acuminata* complex were the dominant *Dinophysis* species in the plankton (up to 1140 cells L^−1^), being likely the main source of DSTs in the area. A second *Dinophysis* species, *D. caudata*, occurred in 2013 when PTX-2 and PTX-2 seco acid were detected in plankton and mussel samples.

Since both *O. vulgaris* and DST-producing dinoflagellates are distributed worldwide, toxin accumulation in octopuses is expected to occur in other regions of the globe. Therefore, octopuses can be considered a potential vector of DSTs to both humans and top predators such as marine mammals, representing a risk for diarrheic shellfish poisoning (DSP) outbreaks and perhaps other sub lethal negative effects upon recurrent exposure. The capacity for toxin transfer and its retention into edible tissues must be better investigated in octopuses in order to assess whether this important fishing product represents a risk for human health in areas subjected to recurrent toxic algal blooms.
